# Electrocortical Dynamics in Children with a Language-Learning Impairment Before and After Audiovisual Training

**DOI:** 10.1007/s10548-015-0466-y

**Published:** 2015-12-15

**Authors:** Sabine Heim, Naseem Choudhury, April A. Benasich

**Affiliations:** Center for Molecular and Behavioral Neuroscience, Rutgers University-Newark, 197 University Avenue, Newark, NJ 07102 USA; School of Natural Sciences & Psychology, Liverpool John Moores University, Byrom Street, Liverpool, L3 3AF UK; School of Social Science and Human Services, Ramapo College of New Jersey, 505 Ramapo Valley Road, Mahwah, NJ 07430 USA

**Keywords:** Auditory sequential processing, Computerized training, Electroencephalography (EEG), Event-related potential (ERP), Specific language impairment (SLI)

## Abstract

Detecting and discriminating subtle and rapid sound changes in the speech environment is a fundamental prerequisite of language processing, and deficits in this ability have frequently been observed in individuals with language-learning impairments (LLI). One approach to studying associations between dysfunctional auditory dynamics and LLI, is to implement a training protocol tapping into this potential while quantifying pre- and post-intervention status. Event-related potentials (ERPs) are highly sensitive to the brain correlates of these dynamic changes and are therefore ideally suited for examining hypotheses regarding dysfunctional auditory processes. In this study, ERP measurements to rapid tone sequences (standard and deviant tone pairs) along with behavioral language testing were performed in 6- to 9-year-old LLI children (n = 21) before and after audiovisual training. A non-treatment group of children with typical language development (n = 12) was also assessed twice at a comparable time interval. The results indicated that the LLI group exhibited considerable gains on standardized measures of language. In terms of ERPs, we found evidence of changes in the LLI group specifically at the level of the P2 component, later than 250 ms after the onset of the second stimulus in the deviant tone pair. These changes suggested enhanced discrimination of deviant from standard tone sequences in widespread cortices, in LLI children after training.

## Introduction

The ability to detect and discriminate change in the auditory environment is crucial for a wide spectrum of behavioral and cognitive processes. Research across the past decade has demonstrated that the ability to detect subtle sound changes early in infancy is highly associated with efficient acquisition of language skills (e.g., Benasich et al. 2002; Choudhury and Benasich 2011; Kuhl et al. 2008; Tsao et al. 2004). Specifically the fast sequential changes in amplitude and frequency related to speech require rapid analysis on the level of sensory processing (bottom-up), and then require identification and isolation (top-down) from competing simultaneous sounds, such as environmental noise. This complex auditory task is achieved with ease and in a seemingly effortless fashion over typical development, but is believed to go awry in a condition termed language-learning impairment (Tallal and Gaab 2006). One approach that allows analysis of the critical auditory dynamics that may be dysfunctional in childhood language disorders is to implement a training protocol thought to impact multilevel auditory processing and observe whether there is a relative change upon completion of the protocol. Here we use event-related potentials (ERPs) to examine the brain correlates of dynamic changes following audiovisual training in children with a language-learning impairment, compared to a no-treatment group of typically developing peers.

In human neuroscience research, auditory processing is often studied using ERPs extracted from the ongoing electrophysiological activity in the electroencephalogram (EEG). Auditory ERPs assess the neural mass activity that is related to an acoustic event, such as a tone or spoken word, and are obtained by averaging across many trials of the same type of event. The resulting ERP waveform consists of positive (P) and negative (N) going voltage deflections, which may vary as a function of the stimulus used and the task. Reliably recurring deflections, sometimes referred to as components, are typically labeled by their polarity and temporal position within the waveform, such as the P1, N1, P2, and N2 components. The temporal unfolding of ERP components is thought to reflect the cascade of electrocortical processes associated with processing the time-locked stimulus event. Thus, ERPs are particularly suitable to examine temporal dynamics associated with different stimuli or tasks. Variations in the amplitude, latency, or topography across the scalp of a given ERP component are often used to explore and quantify changes in electrocortical processes as a function of experimental manipulations, or as a function of inter-individual differences. For instance, one classical manipulation involves the oddball paradigm in which a constant sound train is occasionally interrupted by a deviant sound. In terms of ERP effects, this manipulation has been shown to elicit the so-called mismatch negativity (MMN). The auditory MMN is typically measured as a difference waveform by subtraction of the deviant-minus-standard ERP (for a review see Näätänen et al. 2007) and will be reviewed in the following paragraph. It is reliably seen in passive listening conditions and thus does not critically depend on the participants being engaged in a task (cf., Näätänen et al. 2007; Sussman et al. 2014). Conceptually, the MMN is thought to represent a change detection response of the brain, based on a comparison of the deviant sound to a memory trace of the frequent event. Many types of sound manipulations have been found to elicit an MMN, ranging from frequency, intensity, duration, and spatial location, up to higher-order violations of abstract regularities, such as omitting the second tone of two paired tones (Näätänen et al. 2007). In adults, the MMN tends to occur in a time window ranging from around 150–300 ms after stimulus onset with a maximal negativity over fronto-central scalp sites. Although the MMN can be observed in neonates and seems morphologically comparable to the adult response around 9 years of age (e.g., Hämäläinen et al. 2008; Vestergaard et al. 2009), amplitude of the MMN has been found to increase from preadolescence into adulthood (Bishop et al. 2011). Notably, MMN latencies of 150 to 300 ms have also been observed in children (e.g., Oades et al. 1996). The amplitude, polarity, and topography of the MMN have been described as changing through childhood and adolescence (Segalowitz et al. 2010; Wetzel et al. 2006), with increasing age associated with greater sensitivity to change. Of note for the present study, MMN in children has been shown to display more lateralized topography compared to adults (Martin et al. 2003). In addition, several authors report on a mismatch positivity, especially in children younger than 6 years (e.g., Dehaene-Lambertz and Gliga 2004; Maurer et al. 2007; Shafer et al. 2010). Of course, the polarity of the mismatch response at a given site will be heavily influenced by the reference montage, the number of sensors, and the algorithms used for mapping/interpolation in cases where dense-array electrode systems are employed. The current study combines dense-array recordings with a conservative source density mapping strategy to explore the cortical surface distribution of electrocortical potentials, including the MMN.

In addition to the extensively studied MMN component, earlier deflections of the auditory ERP, often called obligatory components, have also been shown to be sensitive to manipulations such as stimulus regularity or experimental task. These components are typically extracted from the non-difference waveforms of the ERP in different experimental conditions. For instance, Ruhnau et al. (2011) found evidence that random versus repetitive presentation of simple tones modulated the fronto-central N1 at 90–130 ms after tone onset (relative response enhancement in the random condition) in adults, and similarly in 9- to 10-year-old children, both with respect to amplitude and latency levels. Further auditory ERP components, such as the P1 over fronto-central sites, often observed at earlier latencies of around 50–80 ms in adults and 70–100 ms in preadolescents, indicated mixed results with respect to task-modulation. Differences may arise across development, with older participants showing early (P1) amplitude effects of stimulus repetition not seen in children (Ruhnau et al. 2011). Developmental divergence in amplitude modulation has also been reported for the later P2 deflection in a paradigm comparing attend (discriminating between short and long sounds with occasional distraction by pitch variations) and ignore (watching a silent video while ignoring the aforementioned set of sounds) conditions (Wetzel et al. 2006): In a time frame of 168–208 ms post-stimulus onset, 6- to 8-year-olds were found to exhibit a larger frontal P2 in the attend than ignore condition, while adults evinced the opposite pattern. No such modulation effects were seen in early adolescents aged 10–12 years. The present study builds on this body of work, examining both the MMN and non-difference, obligatory components, namely the P1, N1, and P2 components of the ERP.

Childhood language impairments can occur for manifold reasons and rank among the most prevalent of all developmental disorders. It is estimated that about 7 % of all kindergarten children exhibit significant language learning delays of unknown etiology (Tomblin et al. 1997)—a condition often termed “specific language impairment” (SLI). SLI has a hereditary component and is characterized by difficulties in understanding and/or producing speech in the context of unremarkable sensory, non-verbal cognitive, physiological, and socio-communicative development, as well as adequate instruction (Leonard 2014; see Bishop 2014 for a recent discussion). It seems likely that the precursors of SLI can be observed very early in life, but also that language deficits persist, usually in subtle form, into later childhood, adolescence, and conceivably into adulthood. Children with SLI are at higher risk for reading failure (or even developmental dyslexia) and other academic achievement difficulties, school dropout, as well as social and emotional problems (Heim and Benasich 2006). In recent years, the term language-learning impairment (LLI; Tallal and Heim 2015) has become increasingly popular among researchers, acknowledging that language-learning disturbances, across development, often affect both spoken and written language, and may co-exist with more general learning problems or other developmental disorders. Thus we will use this term throughout.

Electrophysiological (EEG/ERP) research in childhood language impairments has been productive, and has converged to demonstrate atypical neural activity in LLI compared to control samples, in response to a wide range of auditory stimuli. Reflective of the rich temporal and spatial structure of EEG/ERP data, this work has examined various features of the electrophysiological response, such as different obligatory ERP components, the MMN, neural oscillations, or a combination of these in the context of paradigms challenging different aspects of auditory processing. For example, several studies indicated that individuals with LLI exhibited atypical ERP responses in a time window of around 100–230 ms, when passively listening to tone or speech stimuli, characterized by attenuated amplitudes or changes in morphology (Bishop et al. 2007, 2012; McArthur and Bishop 2004, 2005). Pihko et al. (2008) used the method of magnetic-source imaging and found the magnetic equivalent of the early P1 deflection evoked by repetitive speech syllables to be weaker among LLI children, an effect localized to supratemporal auditory cortices. Parameterizing the MMN in this population, a number of studies revealed amplitude reductions specifically for speech contrasts (for reviews see Bishop 2007; Näätänen et al. 2014). Davids et al. (2011) extended their findings to non-linguistic sweeps precisely matching the spectro-temporal variation of speech sounds and observed generally mitigated MMNs in 5-year-olds with LLI. Together, such data are suggestive of reduced neural sensitivity to rapid spectro-temporal changes in LLI. In this vein, Bishop and McArthur (2004) reported that adolescents with LLI demonstrated less separation of the electrophysiological responses (in the N1-P2 region, measured at frontal electrodes) to tone doublets presented in rapid sequence, compared to typically developing controls. Comparable evidence in school-age children with and without LLI is suggested by a study capitalizing on early (45 and 75 ms post-stimulus onset) sensory oscillations in the gamma range, centered around 40 Hz (Heim et al. 2011): Oscillatory gamma activity was found to be identical across groups for the first of two fast-rate tones, but LLI children showed substantially reduced spectral amplitude and temporal stability of the sensory response for the second tone. This was taken to indicate that in LLI, neural masses in auditory cortex fail to be engaged in a well-synchronized fashion, when rapid processing of acoustic events is needed. Similar electrophysiological research and findings as briefly reviewed here with respect to LLI were also documented in populations with disturbances in written language skills and developmental dyslexia (Nagarajan et al. 1999; see Schulte-Körne and Bruder 2010 for a review).

Electrophysiological studies of sensory cortices in animals have supported the notion that functional brain circuits are shaped by experience and can be altered through specific, temporally cohesive training regimens (cf., Buonomano and Merzenich 1998; de Villers-Sidani and Merzenich 2011). This approach has led to the design of neuroplasticity-based remedies, which assume that the temporal precision of neural coding can be enhanced by intense training in an optimal learning environment. Fast ForWord Language^®^ (FFW) is a computerized adaptive intervention program to impel neuroplastic changes, particularly in auditory temporal dynamics underlying elementary school language skills (Scientific Learning Corporation 2001). In the first series of studies (Merzenich et al. 1996; Tallal et al. 1996), children with LLI not only exhibited an acceleration in auditory rate processing after completion of FFW, but benefitted also in speech discrimination and receptive language skills. Subsequent work in dyslexia revealed concurrent improvements in the literacy domain, as well as changes in metabolic brain activity (Gaab et al. 2007; Temple et al. 2003). A recent meta-analysis, considering work with the most stringent research designs (randomized controlled trials), however, concluded that FFW is not an effective treatment for language problems (Strong et al. 2011). It should be noted that the current study cannot address the question of treatment efficiency, but uses FFW as a means to examine large-scale neural changes that accompany an intervention that takes the form of regular training directed at auditory skills.

The research described here leverages the ability of intervention studies in LLI to examine potential effects of spectro-temporal dynamics on neurocognitive variables encompassing ERPs as well as language skills. Previous work using similar research designs has yielded mixed results. For instance, Pihko et al. (2007) used a training protocol with a focus on improving articulation, phonological, linguistic and rapid processing skills over a period of 8 weeks (20–30 min of time 3 days/week) in preschool children with LLI. Post-intervention, the LLI children were reported to show an increase in response strength of the magnetic P1 and MMN elicited by speech syllables, accompanied by superior discrimination abilities at the behavioral level. Capitalizing on the shaping of auditory discrimination skills in school students with impairments in oral and/or written language, McArthur et al. (2010) observed training-related gains in behavioral performance, which did not manifest in changes of atypical ERPs in the N1-P2 region to tones, phonemes, and syllables. Recently we explored the extent to which early oscillatory responses in auditory cortex, evoked by fast-rate tone doublets, change after audiovisual training in school-age children with LLI (Heim et al. 2013). Behaviorally, improvements on measures of language were observed following completion of training. Pre-intervention we found reduced amplitude and temporal stability of brain oscillations in the gamma range for the second stimulus of a tone doublet. Amplitude reduction for the second tone was no longer evident for the LLI children post-intervention, although these children continued to exhibit degraded temporal stability of the sensory response. ERPs are ideally suited to complement these results that focused on sensory oscillatory activity, by providing a time-domain representation of the auditory response as it unfolds over time. To fully utilize the brain dynamics captured by the ERP signal, we examine both aspects of the difference waveform between standard and deviant sounds, as is customary in MMN research, and aspects of obligatory components such as the auditory P1, N1, and P2.

Capitalizing on the ERP technique, we follow up on the research summarized above using the same sample as in Heim et al. (2013), examining sequence processing of tone pairs in LLI children before and after a treatment intervention as well as a non-treatment group of children with typical language development (TLD) that were also tested at a similar interval. Although this study design does not permit drawing conclusions in terms of causal contributions of a specific intervention, adding a non-treatment group allowed us to evaluate effects not related to the intervention, such as short-term maturational/developmental changes and the consequences of retesting on ERP responses and behavioral performance. Using this approach we investigated the extent to which early ERP components evoked by tone pairs demonstrated sensitivity to LLI status and intervention. In this context, it is important to note that ERP responses observed after the second of two temporally proximal tones likely reflect a superposition of the responses to the first and the second tone. To avoid confusion, we refer to ERP components as N1, P2 etc., based on their temporal position relative to the second tone. This also reflects the fact that in the present design, only the second stimulus of a tone pair may have the function of the deviant, whereas the first tone was always the same. Given that standard-deviant tone pair stimuli have not been extensively studied using ERPs, this study was framed in an exploratory fashion, with appropriate control of alpha accumulation and multiple testing. As an overarching hypothesis, auditory ERP amplitude was expected to differ before versus after the training intervention in the LLI group, potentially reflecting training-related improvements in language functioning.

## Materials and Methods

### Study Participants

A total of 33 children between the ages 6 and 9 years (average age 8.11 years) with English as the primary language volunteered in the present research. To be included in the study, participants had to meet the following criteria: A nonverbal intelligence score of at least 85 as indicated by the performance intelligence quotient (IQ) of the Wechsler Abbreviated Scale of Intelligence (WASI; The Psychological Corporation 1999), normal hearing, no psychotropic medication use, and no diagnosis of neurological illness (e.g., brain injury, epilepsy), autism spectrum disorder, or any other serious psychiatric disease (e.g., depression, anxiety).

Twenty-one children (6 girls) with a formal diagnosis of language impairment constituted the LLI group. All of them were ascertained from private speech and language services in the metropolitan New York area and throughout New Jersey. The LLI participants obtained overall Core Language composites less than or equal to 85 (≤16th percentile) in the Clinical Evaluation of Language Fundamentals—Fourth Edition (CELF-4; Semel et al. 2003), or had at least three CELF-4 standard subtest scores less than or equal to 8 (≤25th percentile) given in the context of language therapy within the last 6 months. The latter feature accommodated the inclusion of children with a formal diagnosis of language impairment receiving comprehensive treatment, who have low performance in some linguistic skills and average performance in others. Psychometric analyses of the internal consistency of the CELF-4 have shown satisfactory Cronbach’s alpha values for subtests, ranging from 0.69 to 0.91, and very good consistency for composite scores ranging from 0.87 to 0.95. Similarly, test–retest reliability coefficients for composite scores ranged between 0.88 and 0.92. Importantly, specificity and sensitivity of the CELF threshold for language problems set at 1 standard deviation (SD) below the mean, vis-à-vis clinical diagnosis of language disorder, were determined as 0.82 and 1.00 (Pearson Education 2008; Semel et al. 2003).

Twelve control participants with TLD (6 girls) were matched by chronological age (see Table 1). They were recruited through local New Jersey schools as well as pediatric practices in Northern New Jersey. The TLD children had no history or family history of language disturbances and yielded overall CELF-4 language composites greater than or equal to 87 (≥19th percentile). In addition, the children had unremarkable pre- and perinatal circumstances, were born full-term and of normal birth weight.

**Table 1:** Demographic characteristics and behavioral assessment scores (presented as standard scores) of the two groups of children during their first visit to the laboratory

	TLD (n = 12)	LLI (n = 21)	*t* value	*p* value
Age (years)	8.24 (0.92)	8.04 (0.95)	0.58	<0.569
Birth weight (g)^a^	3364.25 (608.23)	3458.05 (832.65)	−0.34	<0.738
Gestational age (weeks)^a^	39.83 (0.58)	39.15 (3.13)	0.74	<0.464
Familial SES^b^	57.88 (6.66)	52.43 (9.42)	1.76	<0.088
Maternal age (years)	39.92 (3.55)	42.19 (3.56)	−1.77	<0.088
Maternal education level^c^	6.25 (0.62)	5.90 (0.83)	1.25	<0.221
CELF-4				
Core language	111.25 (12.87)	79.95 (12.97)	6.69	<0.001
Receptive language	111.33 (11.97)	82.33 (10.51)	7.25	<0.001
Expressive language	111.25 (11.16)	80.95 (13.50)	6.58	<0.001
WASI performance IQ^d^	108.67 (13.16)	101.48 (13.50)	1.49	<0.148

Means (SDs) are shown; all *p* values are 2-tailed with a significance level set to 5 %

^a^Information unknown in one LLI participant

^b^Familial SES is based on the Hollingshead Four Factor Index of Social Status (Hollingshead 1975). A mean score of 57.88 falls within the social stratum of major professional (55–66), while 52.43 corresponds to the minor professional category (40–54)

^c^Maternal education level ranging from 1 to 7 according to the Hollingshead criteria. A value of 5 represents partial college, while 6 indicates college/university graduation

^d^In two TLD children the Abstract Visual Reasoning cluster of The Stanford-Binet Intelligence Scale, fourth edition (Thorndike et al. 1986) was used as a WASI Performance IQ equivalent

Basic demographic information is listed in Table 1, together with the language and cognitive achievement scores for the TLD and LLI children. Participant groups did not significantly differ in terms of birth weight, gestational age, familial socioeconomic status (SES), maternal age, and maternal education level. Consistent with their difficulties, LLI children demonstrated, on average, significantly lower overall language performance (CELF-4 Core Language) than TLD children. This was also evident in the areas of Receptive and Expressive Language abilities. All participants scored in the average or above-average age range on the WASI Performance scale, with no significant group differences in nonverbal intellectual functioning (see Table 1).

### Study Protocol

Our University’s Institutional Review Board approved the study protocol. Written informed consent was obtained from all parents of the child participants; children provided oral assent after the project was explained in age-appropriate lay language. Each participant underwent cognitive and language assessment (see “Study Participants” section), as well as electrophysiological testing spread across 2 days during his/her initial visit period (Visit 1). Subsequently, the LLI group participated in the audiovisual training program, which was provided off-site. One to four weeks after completing the training program, children returned to the laboratory for post-intervention behavioral and electrophysiological sessions (Visit 2). Again the sessions took place on two separate days and were identical to Visit-1 testing except that the WASI performance IQ was not reassessed. This decision was based on the finding that practice effects on the performance subtests are greater than on the verbal scale and may only decrease significantly after a 1- to 2-year interval (Matarazzo 1972; Matarazzo et al. 1980). The average number of days between the LLI children’s first and second visits were 116 days (SD = 45) for the behavioral and 102 days (SD = 42) for the electrophysiological assessment.

The TLD group was also tested twice, but did not participate in any intervention program in the interim. On average 92 (SD = 37) and 95 (SD = 53) days elapsed between the Visit-1 and Visit-2 behavioral and electrophysiological assessments. Non-paired *t* tests revealed no significant differences in the Visit-1 to Visit-2 intervals for the two groups of children, *t*s(31) = −1.60 and −0.40, *p*s < 0.120 and 0.691 for the behavioral and electrophysiological sessions, respectively. Thus, the TLD children served as a control for changes on the behavioral and neuronal level related to factors other than the training regimen, such as repeated assessment (e.g., familiarization with the testing environment, practice effects) as well as short-term developmental and maturational effects.

#### Computerized Audiovisual Training

The FFW approach is a computerized intervention program designed to develop core elementary school language skills (Scientific Learning Corporation 2001). In particular, FFW is thought to enhance the rate of auditory sequential processing, aspects of attention and working memory, as well as phonological processing and grammatical skills (Tallal 2004). The software comes in seven visually appealing exercises and includes acoustic events that range from frequency sweeps, to phonemes, to words, up to the sentence level: The objective of Circus Sequence is to indicate the temporal order of two frequency-modulated tones (upward or downward gliding in frequency) at a specified interstimulus interval (ISI). Children are asked to duplicate the sequence of the two sweeps (up-up, up-down, down-up, down-down) by mouse clicking on the element on the computer screen. Old MacDonald’s Flying Farm involves detecting individual phoneme changes in repeated consonant-vowel syllables (e.g., /do/…/do/…/to/). In this exercise, the child is invited to capture a flying animal by using the computer mouse, clicking and holding the button down until he or she hears a sound change, and then releasing the button and thus the animal. The objective of Phoneme Identification is to identify a target phoneme in one of two contrasting consonant-vowel or vowel-consonant-vowel combinations (such as /bi/-/di/ or /aba/-/ada/, respectively). After the child has listened to a target sound, he or she hears two sounds produced sequentially by two characters, and indicates via mouse click which character uttered the target. Phonic Match requires matching syllable pairs in simple words (e.g., /pack/-/pat/). When a tile in a grid is clicked, the child hears a word and has to find the second tile that hides the same sound. Phonic Words involves discrimination between words that differ only by an initial or a final consonant sound (such as /bee/-/knee/ or /run/-/rung/, respectively). The child listens to a word introduced by the prompt “point to” and then clicks on the correct representational image of the word, choosing from the picture pair (e.g., bee vs. knee) presented. Block Commander focuses on following commands of increasing length and grammatical complexity (e.g., “Touch the green square!” or “After touching the yellow square, touch the blue circle!”). Children perform their answers via the computer mouse on a board game shown on the screen. Finally, Language Comprehension Builder aims at training each rule of English grammar, such as negation (e.g., “The baby is not crying.”) or clefting (e.g., “It’s the girl that the boy pulls.”). The child is asked to click on the picture that matches the sentence he or she just heard.

All children with LLI received the intervention regimen under the guidance of a certified provider, who was a licensed speech and language pathologist. Children trained either at the FFW provider’s office or at home following the same administration procedure. Each participant was seated in front of a computer screen where the visual stimuli were shown (e.g., a circus or a farm theme), and accompanying tonal and linguistic sounds were delivered via headphones. Early FFW training utilized acoustic events in which rapid transitions were prolonged in time and differentially amplified. As a child progressed through the exercises and performance improved, the modified acoustic stimuli were presented at rates and amplitude levels closer and closer to those that occur in natural speech. Participants responded via mouse clicks at appropriate locations in the visual array. Feedback was provided on a trial-by-trial basis for the delivered responses: Correct responses were rewarded, for instance, in terms of point gains or auditory/visual animations, incorrect clicks were communicated by an extra auditory cue and by indicating the correct answer prior to the next trial. The presentation of trials in each exercise was self-paced based on an individual child’s skill level. The adaptive algorithm of the software ensured that each participant responded correctly approximately 80 % of the time. This is an important principle common to many neuroplasticity-based programs, in order to provide a heavy dose of correct trials and positive reinforcement (e.g., Tallal et al. 1998; Wilson et al. 2006).

The FFW administrators monitored children’s training participation and progress daily. On each training day, participants’ performance scores from the exercises were uploaded over the Internet to Scientific Learning Corporation and then returned to the provider as a detailed progress report. Completion of the program was confirmed for each student by the provider and the company’s final level report. The LLI group trained about 100 min daily, 5 times a week for an average of 32 days (SD = 12).

#### Electrophysiological Assessment

##### Stimuli and Procedure

In the electrophysiological Visit-1 and Visit-2 sessions, the two groups of children were exposed to complex tones, having a fundamental frequency of 100 or 300 Hz with 15 harmonics (6 dB roll-off per octave). Tones were 70 ms in duration (rise and fall times of 5 ms) and were delivered in pairs separated by a 70-ms ISI (tone onset to onset). A presentation rate of 70 ms falls within the “tens of milliseconds range” (Tallal et al. 1993) that is critical for accurate speech perception and comprehension: Many acoustic cues occur in parallel and/or in rapid succession within syllables and words, such as formant transitions (maximum ca. 80 ms) and voice-onset times (discrimination range ca. 25–70 ms) inherent to stop consonants, or brief formants of short vowels (Borden and Harris 1980; Kewley-Port 1982; Phillips 1999). In their seminal work, Tallal and Piercy (1973a, b, 1974, 1975) found that LLI children displayed low temporal sensitivity to both nonverbal and verbal acoustic changes, presented in the tens of milliseconds range. Specifically, these children required a gap of ≥305 ms in order to accurately sequence two successive 75-ms tones, and were able to discriminate the stop-consonant syllables /ba/ and /da/ when the formant transitions were synthetically extended to 95 ms, but not at 43 ms, i.e., near the natural speed of speech. Comparable findings have been reported at the neural level indexed by the MMN: Children diagnosed with a wider spectrum of learning problems exhibited attenuated MMN responses to short relative to artificially lengthened transition syllables, as well as compared to the responses in typically developing age controls (Bradlow et al. 1999). Similarly, children with LLI were found to show mitigated MMNs to brief (50-ms) vowel contrasts (Shafer et al. 2005) versus longer (250-ms) phoneme exemplars (Datta et al. 2010). Further, a series of studies examining rapid auditory changes in infants suggests that the ability to resolve a 70-ms ISI predicts language outcome at later ages regardless of family history for LLI (e.g., Benasich et al. 2002, 2006; Benasich and Tallal 2002; Choudhury and Benasich 2011).

A stream of 833 tone pairs (tone pairs = trials) was delivered with an intensity of 75 dB free field via speakers to the left and right of the child. The intertrial interval (onset to onset) was fixed at 700 ms. A passive oddball paradigm was used in which the 100-100 Hz tone doublet served as the standard (80 % probability of occurrence: 667 trials), and the 100-300 Hz doublet as the deviant pair (20 % probability of occurrence: 166 trials). A pseudo-random mode ensured that at least three and no more than 10 standards occurred between each deviant. Four regularly placed pauses allowed participants to take a short break.

Children were seated in a comfortable chair in an acoustically shielded room. To control for level of arousal, participants watched silent videos and were asked to ignore the sounds. During the pauses, the experimenter spoke to the children (to ask about fatigue, comfort, etc.) and posed questions about the movie to ensure they were attending to it. Each child was motivated to respond correctly in order to earn stickers (placed on a cut-out shape) needed to “buy” a prize at the end of the session (all participants received a prize at the end of each visit’s session regardless of the number of stickers they earned). In addition, participants were asked to prevent unnecessary eye or body movements during recordings. Compliance was verified by video monitoring.

##### Data Acquisition

The EEG was recorded from 64 sensors using an Electrical Geodesics ™ (EGI; Eugene, OR, USA) system with a sampling frequency of 250 Hz referenced to the vertex (recording site Cz). Impedances were maintained below 50 kΩ, as recommended for the EGI high input-impedance amplifiers (200 MΩ input impedance). Horizontal and vertical electro-oculogram (EOG) was determined from electrodes located at the outer canthi as well as above and below the eyes. All channels were pre-processed online by means of elliptical 0.1 Hz high-pass and 100 Hz low-pass (cut-offs at 3 dB point, respectively) filters implemented in the EGI recording software.

##### Data Reduction

First-step offline analyses were performed by using commercial Brain Electrical Source Analysis software (BESA Research Version 5.3; BESA GmbH, Germany, 2012): Data were arithmetically re-referenced to an average reference configuration and filtered with a Butterworth band-pass filter with a low cut-off (3 dB point) at 1.0 Hz, and a high-cutoff (3 dB) at 40 Hz. Subsequently, data were corrected for ocular artifacts (blinks, vertical, and horizontal eye movements) by applying the algorithm of Ille et al. (2002), which uses temporal and spatial information to identify pre-defined types of artifacts and then applies spatial filters to the data for correction. Epochs were then extracted from the continuously recorded EEG relative to the onset of the tone pair, using a 300 ms pre- and 915 ms post-tone pair window. Single epochs characterized by a signal amplitude, gradient, and variance of the gradient larger than 200, 150, and 0.1 µV, respectively, were excluded as artifacts from the subsequent averaging process. For each participant, artifact-free epochs were averaged by visit (first, second) and tone-pair type (deviant, pre-deviant standard), and the mean voltage of the 100-ms pre-tone doublet segment was subtracted as the baseline. Averaging was limited to the standard occurring prior to a deviant. This procedure ensured (1) that difference waveforms are based on comparable signal-to-noise ratio between the two stimulus types, and (2) that the experimental context (i.e., the nature and temporal structure of the preceding trials) in which the stimulus occurred was also comparable between standards and deviants. The mean number (±SD) of averaged epochs across participants and visits were 126 (±17) for deviant and 125 (±18) for standard tone pairs, and did not differ as a function of group (TLD, LLI) and visit (first, second).

For each participant and tone-pair type, the averaged voltage data from Visits 1 and 2 were interpolated using spherical splines, assuming a spherical volume conductor and then projected to a current source density (CSD) representation, using the algorithm proposed by Junghöfer et al. (1997). This transformation is based on the Laplacian (the second spatial derivative) of the voltage maps for each time point, thus amplifying scalp regions with changing voltage gradients. The CSD is often used as an avenue to heightening the spatial specificity of the voltage map without involving model fitting and parameter estimation, as is the case with source estimation techniques. Thus, this approach assists in capitalizing on the spatial information provided by dense-array EEG (Keil et al. 2014). A smoothing factor of lambda = 2 was selected for spherical spline interpolation (Junghöfer et al. 1997). The complete algorithm is implemented in the open source software package Electromagnetic Encephalography Software (EMEGS Version 2.5; www.emegs.org). CSD data were then grand averaged by tone-pair type (standard, deviant) and visit (first, second) within the LLI and TLD groups.

##### Waveform Analysis: Amplitude

**Fig. 1:**
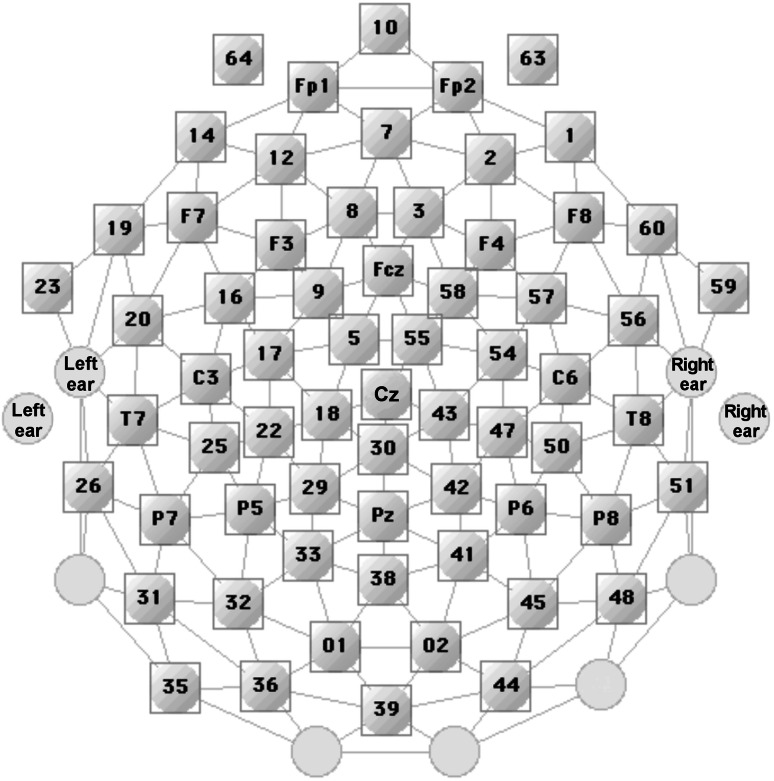
Layout of the sensor array. Frontal electrodes are shown at the top of the figure. Sites roughly corresponding to locations of the international 10–20 system are also depicted (*green*). Different groups of electrodes were formed for each CSD-based ERP component, and voltages averaged within each participant for statistical analyses, as described in the “Materials and Methods” section. Adapted from Net Station Acquisition—Technical Manual by Electrical Geodesics, Inc., 2003 (Color figure online)

**Fig. 2:**
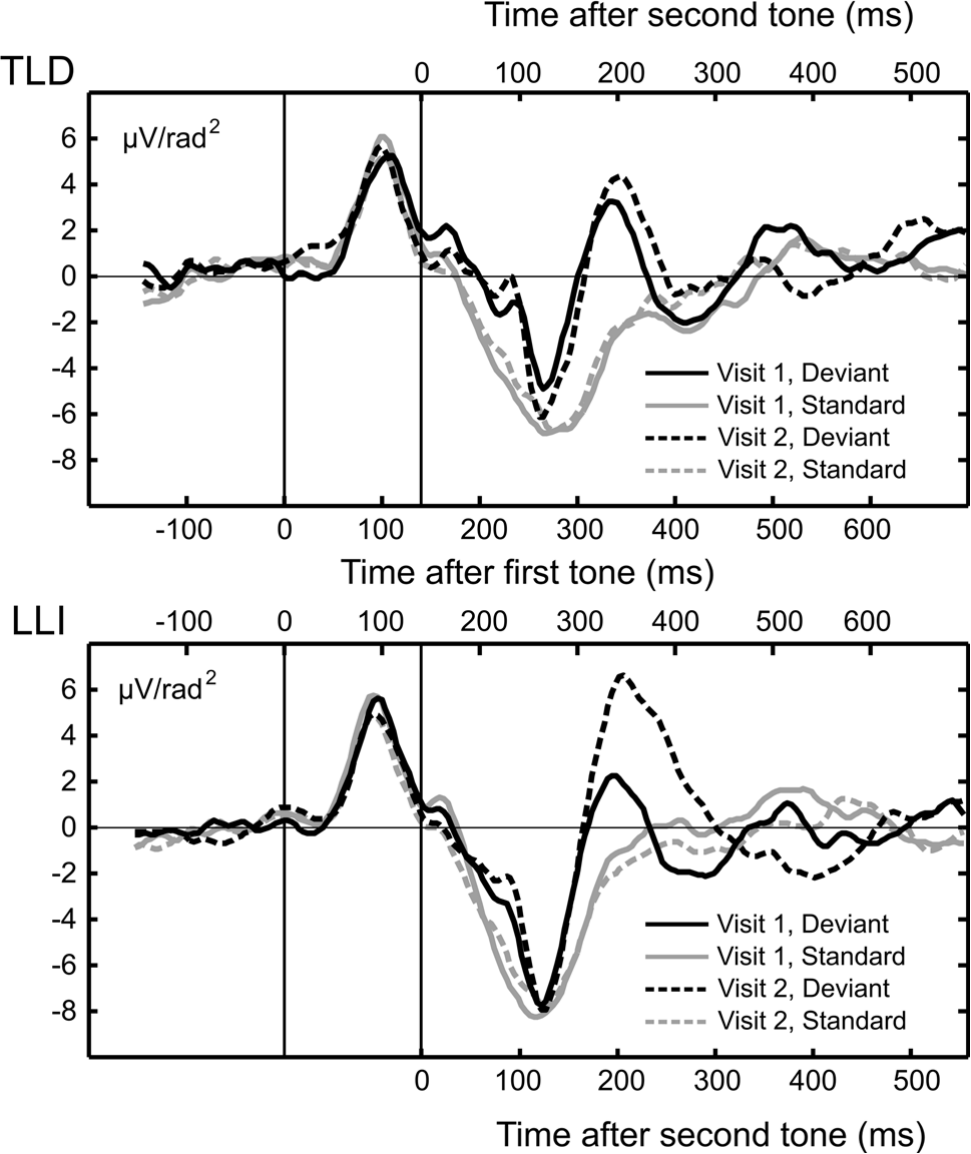
Grand mean CSD waveforms over a representative group of fronto-central sensors (Cz and their nearest anterior neighbors 5 and 55, Fcz and their nearest posterior neighbors 9 and 58) at each visit for the two groups in the study, 12 children with TLD (*top plot*) and 21 children with LLI (*bottom plot*). Waveforms are shown at the latencies (see “Materials and Methods” section for details) of the P1-N1-P2 peaks interspersed by a negative-going deflection in the MMN latency range (following the N1) in response to standard (*gray lines*) and deviant (*black lines*) tone pairs. The inner abscissa in each plot indicates the time scale with respect to the first tone in a doublet, the outer abscissa the time scale with respect to the second tone. At both Visit 1 *(solid lines*) and Visit 2 (*dashed lines*), waveform morphology was similar across study groups. Note the superposition of the deflections evoked by the two subsequent stimuli of each tone-pair type

Given the exploratory nature of the study, temporal areas of interest were defined for each peak of the non-difference waveforms visible at fronto-central sensor locations as shown in Fig. 2. Time windows for analysis were selected to contain the peak amplitude at the scalp region with maximum current source density at the scalp sites of interest as well as to contain temporally adjacent data points of the same polarity, in an electrode cluster of sufficient size (3 sensors or more). Additional time windows were formed for the P2 component, which showed a more complex waveform and displayed differential sensitivity to experimental components for an early segment (containing the peak) and a downward slope (late portion), following the peak. All time windows were selected to maximize the inclusion of comparable electrocortical events across participants in both groups. Please note again in this context that this study aimed to compensate the disadvantages of an exploratory strategy by selecting strong cortical signals that appeared in a robust fashion across children, in terms of time course and topography. Effects of visual inspection and subsequent statistical double dipping were addressed by false discovery rate correction (see below). Furthermore, based on the literature, we identified a difference waveform in the MMN time range, showing a maximum at fronto-lateral electrodes. This component was also included in the pool of exploratory analyses, the results of which were subject to correction for multiple comparisons as described below. Four deflections of the CSD waveforms survived rigorous correction and were reliably present following onset of the second stimulus of the standard and deviant tone pairs across participants: P1 (76–92 ms), N1 (124–140 ms), a negative-going segment indexing the MMN (160–220 ms, see below), and the late period of the complex P2 component (264–280 ms). The latter component showed pronounced variability in latency and complexity, varying strongly with tone-pair type. It was thus examined in terms of an earlier and later period, only the later period of which survived correction for multiple comparisons. The corresponding mean peak latencies for each of these components were 89, 131, 175 ms (peak of the difference waveform in the MMN range), and 210 ms (measured as the overall peak of the complex P2 component), across tone-pair types (see Fig. 2). These latencies are all given relative to the onset of the second stimulus of the tone doublet. Time windows were selected upon visual inspection of the grand-mean topographical distributions at central, lateral, and frontal electrode sites, where the amplitudes were most pronounced. Because the major CSD deflections showed topographies with symmetrical distribution along the midline, a hemisphere factor was not considered in the analyses. Voltage amplitudes were then averaged across the time bins within a specified window, and across the sensors with a given electrode cluster. For the purpose of statistical analysis, one regional mean across symmetrically located electrode sites were formed, covering the area of maximum voltage change in each deflection. P1 included electrode site Fcz and its nearest anterior neighbor sensors 8 and 3, N1 included site Fcz with its nearest posterior neighbors 5 and 55, and P2 encompassed electrode site Cz and its nearest anterior neighbors 5 and 55. For the negative-going segment used to parameterize the MMN, we grouped sites F7 and C3 with their nearest posterior neighbor sensors 16, 20, and 25, respectively on the left, as well as sites F8 and C6 with their nearest posterior neighbors 57, 56, and 50, respectively on the right. The layout of the sensor array is shown in Fig. 1.

##### Waveform Analysis: Latency

To fully use the temporal information inherent in ERP data, we conducted additional exploratory latency analyses, testing the overall hypothesis that electrocortical dynamics changed between visits, in a different fashion for LLI and TLD children. Latency differences between conditions and groups were evaluated by means of *t* tests for electrode groups showing reliable amplitude effects, using the Jackknife method (Kiesel et al. 2008). This approach has been shown to be more sensitive to real latency differences than single-participant-based scoring methods while at the same time being less affected by noise. Jackknife-based statistics involve re-computation of the desired test statistic, leaving out one observation at a time from the sample set. In the present case, we were interested in latency changes of the CSD deflections in the difference waveform [∆ (deviant − standard)] as function of group and visit. The difference wave was used to minimize the number of latency tests. For both the TLD and LLI group, we calculated within-participants Jackknife *t* tests by first forming a set of new averaged waveforms to replace any of the participants’ individual difference waveforms for Visits 1 and 2. Each of these waveforms represented a grand mean across all participants per group but one. From these waveforms, the latency of each event of interest (i.e., each component visible in Fig. 2) was scored as the point in time when 50 % of the maximum (or minimum, for negative-going waveforms) amplitude of that event was reached. Jackknife *t* values were then calculated as the ratio of the grand mean difference in milliseconds, divided by the Jackknife estimate of the standard error of the difference SD, as described in Miller et al. (1998), comparing the latency for every component in the difference waveform between Visit 1 and Visit 2, for each group separately. This analysis resulted in *t* tests indicating whether the latency of a given component changed significantly from Visit 1 to Visit 2, in the TLD or LLI group.

### Statistical Analyses

At the behavioral level, differential changes in language measures from Visit 1 to Visit 2 for the LLI (FFW training) and TLD (no training) groups were analyzed using 2(Group) × 2(Visit) mixed-factors Analyses of Variance (ANOVAs). Dependent variables included CELF-4 Core Language standard score, CELF-4 Receptive Language standard score, and CELF-4 Expressive Language standard score. Post-hoc inspection of significant interaction effects (*p* < 0.05) was effected by contrast analyses. If main effects of Group and Visit without a significant interaction of these factors were identified, paired samples *t* tests were planned to further investigate the changes in language performance from Visit 1 to Visit 2 in each group of children (Heim et al. 2013).

To examine changes in brain-electric activity, the CSD amplitude means of the P1, N1, and P2 components and the negative-going segment indexing the MMN were submitted to mixed ANOVAs crossing the between-participants factor Group (2; LLI, TLD) and within-participants factors Visit (2; 1, 2) and Tone Pair (2; deviant, standard). Contrast analyses were planned to follow up significant interaction effects. To counteract the multiplicity effect across all test statistics on amplitude means, we controlled for false discovery rate (FDR) at the 5 % level by adopting the Benjamini-Hochberg algorithm (Benjamini and Hochberg 1995). This algorithm resulted in a corrected significance level of *p* ≤ 0.019. To explore systematic variations (*p* < 0.05) in the latencies of the ERPs from Visit 1 to Visit 2 as a function of group, a series of paired Jackknife-based *t* tests were performed with the difference waves in the TLD as well as LLI children (see “Waveform Analysis: Latency” section). Again, obtained *p* values were controlled by FDR correction, separate from the amplitude analyses, resulting in a critical *p* value of 0.020 corresponding to an alpha of 0.05. For all analyses run on behavioral assessment and electrocortical amplitude data, we report partial eta-squared (η_*P*_^2^) values as a measure of effect size (Cohen 1988).

## Results

The present research aimed at investigating the extent to which ERPs to tone-pair sequences change after FFW training, designed to improve language skills by enhancing the temporal precision of auditory encoding. In terms of language ability, results of differential change from Visit 1 to Visit 2 in the same sample of children have been reported previously (Heim et al. 2013), but are included here to facilitate reading.

### CELF-4 Language Outcome

Performance scores in the CELF-4 at Visit 2 as well as in relation to their change from Visit 1 for the two groups of children are summarized in Table 2. For all language variables examined, mixed-design ANOVAs yielded significant main effects of Group [Core, *F*(1,31) = 40.89, *p* < 0.001, η_*P*_^2^ = 0.57; Receptive, *F*(1,31) = 36.06, *p* < 0.001, η_*P*_^2^ = 0.54; Expressive, *F*(1,31) = 41.88, *p* < 0.001, η_*P*_^2^ = 0.57] and Visit [Core, *F*(1,31) = 27.87, *p* < 0.001, η_*P*_^2^ = 0.47; Receptive, *F*(1,31) = 15.05, *p* < 0.001, η_*P*_^2^ = 0.33; Expressive, *F*(1,31) = 16.86, *p* < 0.001, η_*P*_^2^ = 0.35]. Overall, CELF-4 scores were superior in TLD than LLI children and higher at Visit 2 than Visit 1. The main effects were qualified by a significant Group by Visit interaction, for both the Core Language composite, *F*(1,31) = 12.02, *p* < 0.002, η_*P*_^2^ = 0.28, and the Receptive Language index, *F*(1,31) = 9.60, *p* < 0.005, η_*P*_^2^ = 0.24. Post-hoc contrast analyses revealed considerable performance gains on both language measures for the LLI group [Core, *F*(1,31) = 52.60, *p* < 0.001, η_*P*_^2^ = 0.63; Receptive, *F*(1,31) = 33.47, *p* < 0.001, η_*P*_^2^ = 0.52], but no systematic variation in the TLD group between visits. With respect to the Expressive Language index, the two-way interaction failed to reach significance, *F*(1,31) = 3.71, *p* < 0.064, η_*P*_^2^ = 0.11. To examine whether there was a differential change from the first to the second visit as a function of treatment, paired *t* tests were conducted within each group. While the LLI children showed evidence of increased expressive language scores, *t*(20) = −4.44, *p* < 0.001, η_*P*_^2^ = 0.50, no significant change from Visit 1 to Visit 2 was seen in the TLD children (see Table 2).

**Table 2:** Language scores (CELF-4 standard scores) during children’s second visit to the laboratory as well as in relation to their change from Visit 1 by participant group

	TLD (n = 12)	LLI (n = 21)
Core language		
Visit 2	112.92 (11.90)	88.00 (11.84)
Difference from Visit 1	+1.67 (4.46)	+8.05 (5.40)*
Receptive language		
Visit 2	112.42 (13.94)	92.00 (12.25)
Difference from Visit 1	+1.08 (6.86)	+9.67 (8.06)*
Expressive language		
Visit 2	113.92 (10.56)	88.33 (12.83)
Difference from Visit 1	+2.67 (4.83)	+7.38 (7.62)*

Means (SDs) are shown; difference scores express the change in CELF-4 standard scores at Visit 2 relative to Visit 1

* Significant increase in standard scores from Visit 1 to Visit 2 (all *p*s < 0.001)

### Evoked Brain Responses to Tone-Pair Events

**Fig. 3:**
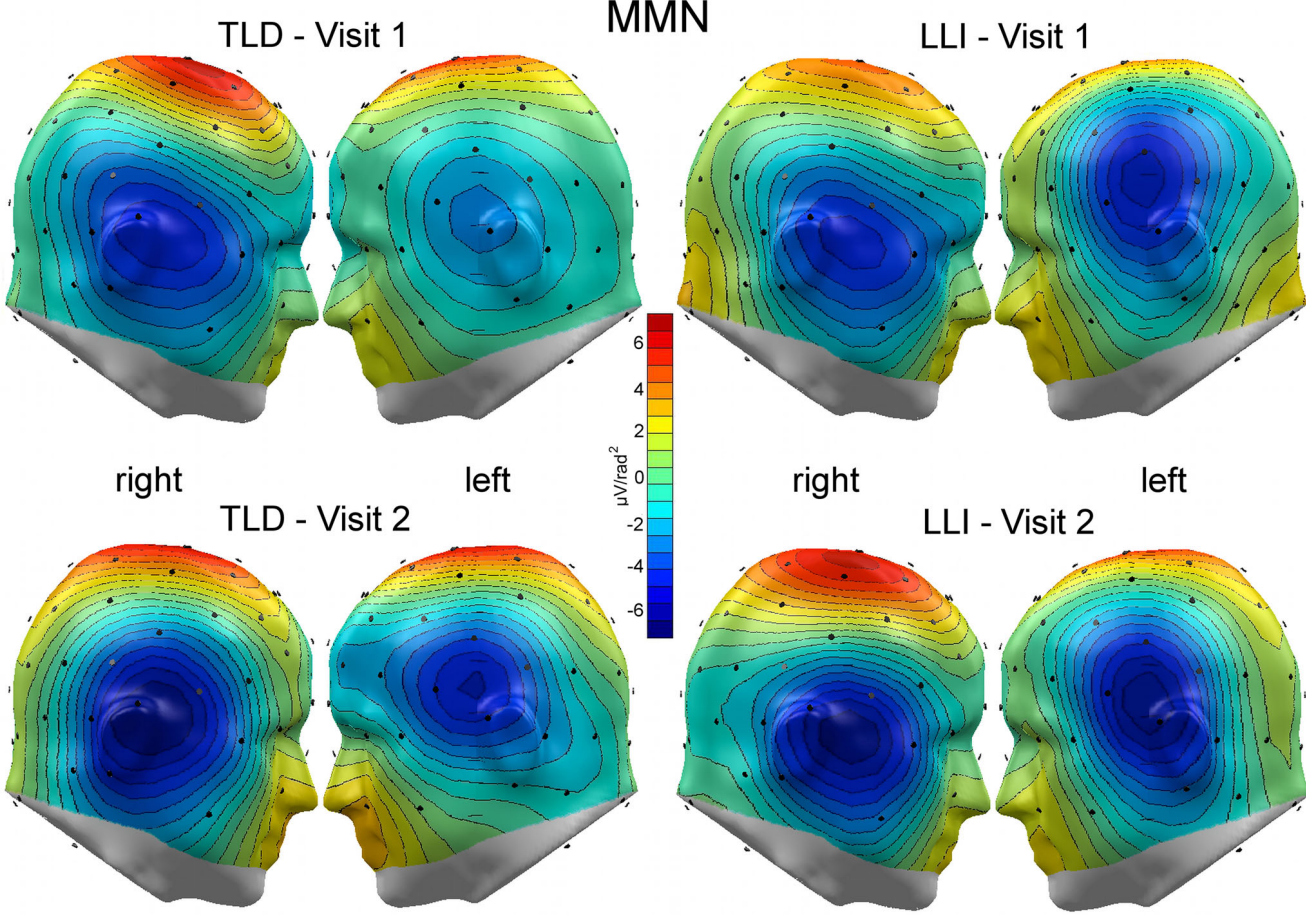
Grand mean spline-interpolated CSD distribution of the difference wave (deviant–standard) during the MMN time range (see “Materials and Methods” section) at each visit for the two groups in the study, 12 children with TLD (*left*) and 21 children with LLI (*right*). Note the consistent topography of the electrocortical response across visits and groups, illustrated in this example (Color figure online)

In both groups of children, time domain averaging resulted in a well-defined pattern of ERP waveforms, showing clear evidence of superposition of responses evoked by the two subsequent auditory events of each tone-pair type (e.g., Bishop and McArthur 2004). This was also evident in the CSD representation of the data, as indicated in the grand mean CSD waveforms over a representative group of fronto-central sensors (see Fig. 2). At both assessment times, waveform morphology was similar across participant groups, representing a P1-N1-P2 complex interspersed by a negative-going deflection in the MMN latency range in response to standard and deviant pairs. The topographies in Fig. 3 illustrate the grand mean spline-interpolated CSD distribution during the MMN window, i.e., the subtraction wave “deviant-standard responses”, for each group and visit. This example further supports the consistency of the electrocortical response across children at Visit-1 and Visit-2 testing.

Mixed-design ANOVA run on P1 amplitude data yielded a significant main effect of Tone Pair, *F*(1,31) = 34.08, FDR corrected *p* < 0.001, η_*P*_^2^ = 0.52, reflecting response enhancement of the deviant stimulus across participant groups and visits (see Fig. 2). No other effects approached statistical significance. A similar pattern of results was observed for the N1 component with deviant tone doublets eliciting overall larger amplitudes than standard doublets, *F*(1,31) = 7.45, FDR corrected *p* < 0.011, η_*P*_^2^ = 0.19 (see Fig. 2). This significant main effect was not qualified by any interaction between Tone Pair, Visit, and Group.

**Fig. 4:**
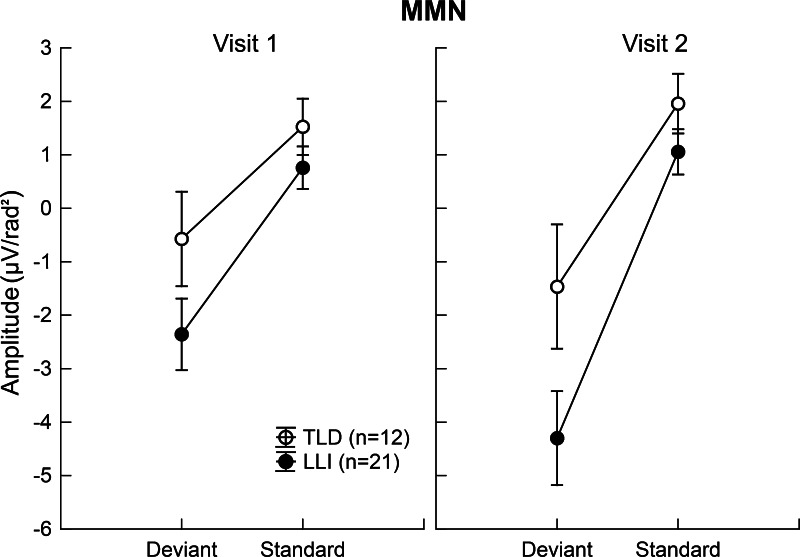
Mean amplitude of the negative-going deflection in the MMN latency range averaged across a subset of fronto-lateral sensors (F7, 16, 20, C3, 25 and F8, 57, 56, C6, 50 on the left and right, respectively) for deviant and standard tone pairs at Visits 1 and 2. Values show means of 12 children with TLD (*open circles*) and 21 children with LLI (*filled circles*). *Vertical bars* reflect standard errors of mean. Typical for a mismatch response, the deviant tone pairs elicited an overall larger negativity than standard pairs in both groups of children. The magnitude of the deviant-related negativity was even more pronounced at Visit 2, compared to Visit 1, and did not vary as a function of group membership

**Fig. 5:**
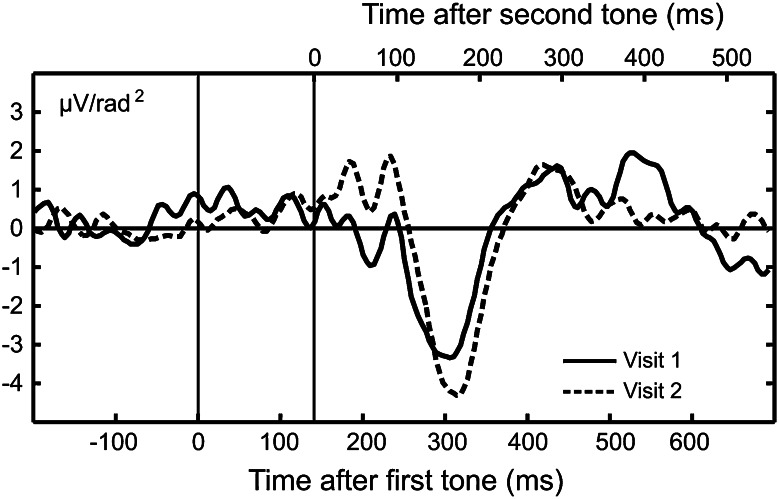
The grand mean (n = 33) CSD deviant–standard difference waveform, for the entire sample, at Visits 1 (*solid line*) and 2 (*dashed line*), averaged across a subset of fronto-lateral sensors (F7, 16, 20, C3, 25 and F8, 57, 56, C6, 50 on the left and right, respectively). The bottom abscissa indicates the time scale with respect to the first tone in a doublet, the top abscissa the time scale with respect to the second tone. Note the pronounced negative deflection in the time range between 160 and 220 ms after onset of the second tone

The ANOVA conducted on the negative-going deflection in the MMN latency range revealed a significant main effect of Tone Pair, *F*(1,31) = 33.47, FDR corrected *p* < 0.001, η_*P*_^2^ = 0.52. Indicative of a mismatch response, the deviant tone doublets elicited an overall larger negativity than standard doublets. This effect was qualified by a significant Visit x Tone Pair interaction across both groups of children, *F*(1,31) = 8.28, FDR corrected *p* < 0.008, η_*P*_^2^ = 0.21 (see Fig. 4). Focused contrasts indicated that the deviant-evoked response underwent a systematic negative enhancement at Visit 2, compared to Visit 1, *F*(1,31) = 8.80, FDR corrected *p* < 0.006, η_*P*_^2^ = 0.22. This supported a more prominent stimulus difference, i.e., MMN, during the second, *F*(1,31) = 33.56, FDR corrected *p* < 0.001, η_*P*_^2^ = 0.52, relative to the first, *F*(1,31) = 19.47, FDR corrected *p* < 0.001, η_*P*_^2^ = 0.39, assessment time. The deviant–standard difference/MMN for the entire sample at Visits 1 and 2 is illustrated in Fig. 5.

**Fig. 6:**
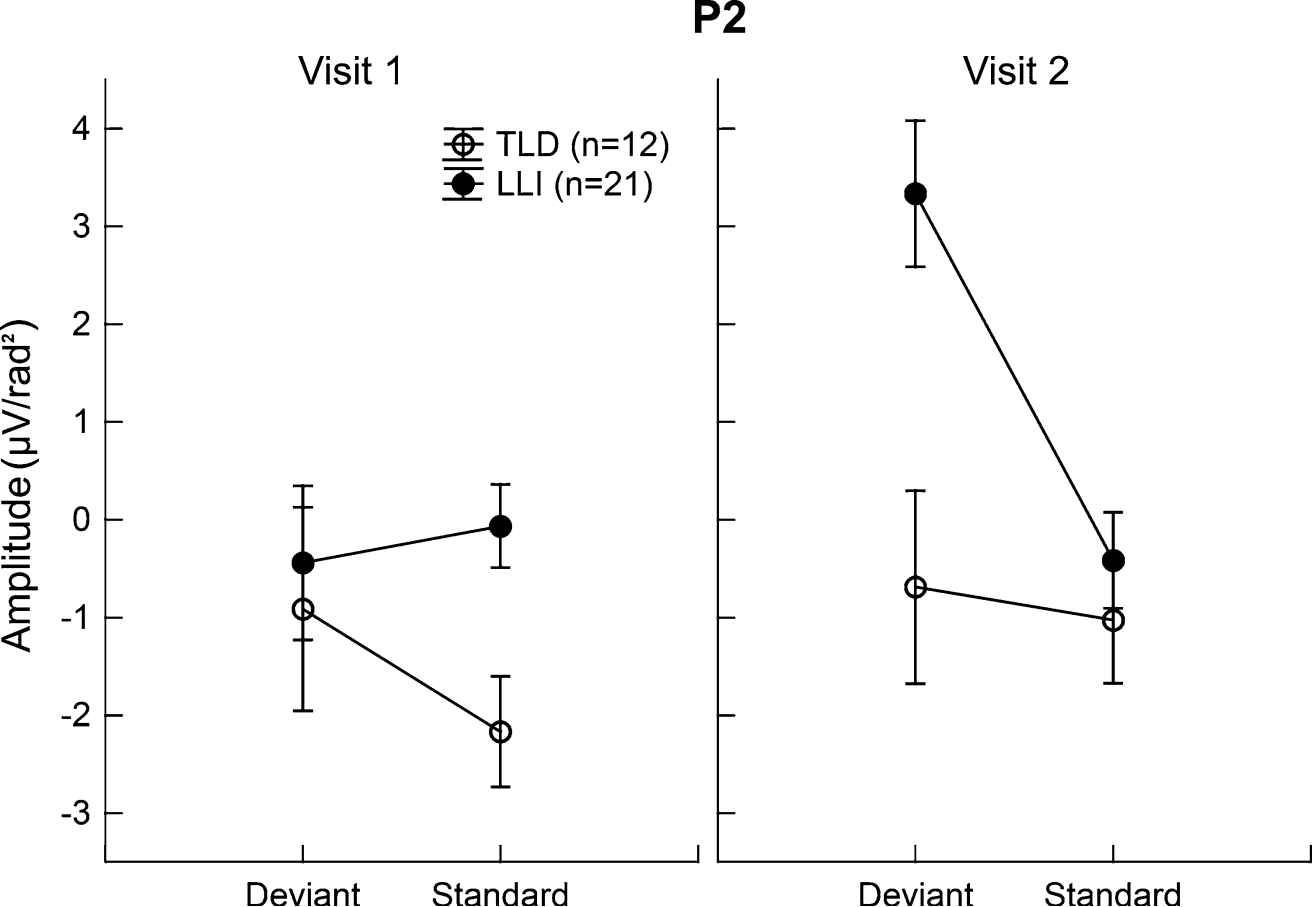
Mean amplitude of the P2 downward slope averaged across a subset of fronto-central electrode sites (Cz, 5, and 55) for deviant and standard tone pairs in the two groups of children at Visits 1 and 2. Values represent means of 12 children with TLD (*open circles*) and 21 children with LLI (*filled circles*). *Vertical bars* indicate standard errors of mean. There were no systematic variations in the P2 amplitude across visits in the TLD group, but the LLI group showed a pronounced amplitude increment for the deviant tone doublet from Visit 1 to Visit 2. This induced a significant group difference at Visit 2 indicating stronger deviant evoked responses in LLI than TLD children

With respect to ANOVA results of the P2, mean amplitudes of the late portion were significantly larger in LLI than TLD children, *F*(1,31) = 8.02, FDR corrected *p* < 0.009, η_*P*_^2^ = 0.21, and more pronounced at Visit 2 than Visit 1, *F*(1,31) = 10.07, FDR corrected *p* < 0.004, η_*P*_^2^ = 0.25. These main effects were modified by a significant Group × Visit × Tone Pair interaction, *F*(1,31) = 6.87, FDR corrected *p* < 0.014, η_*P*_^2^ = 0.18 (see Fig. 6). Focused contrasts revealed that at Visit 1, the standard evoked P2 was significantly larger in LLI than TLD children, *F*(1,31) = 8.83, FDR corrected *p* < 0.006, η_*P*_^2^ = 0.22. Furthermore, there was evidence of differential change across visits: The LLI group, but not the TLD group, showed an increase in amplitude for deviant tone pairs from Visit 1 to Visit 2, *F*(1,31) = 21.45, FDR corrected *p* < 0.001, η_*P*_^2^ = 0.41. Consequently, at Visit 2, the LLI children’s response magnitude for deviants was not only greater relative to their response for standards, *F*(1,31) = 15.08, FDR corrected *p* < 0.001, η_*P*_^2^ = 0.33, but also exceeded the deviant-related amplitude size of TLD children, *F*(1,31) = 10.56, FDR corrected *p* < 0.003, η_*P*_^2^ = 0.25.

**Fig. 7:**
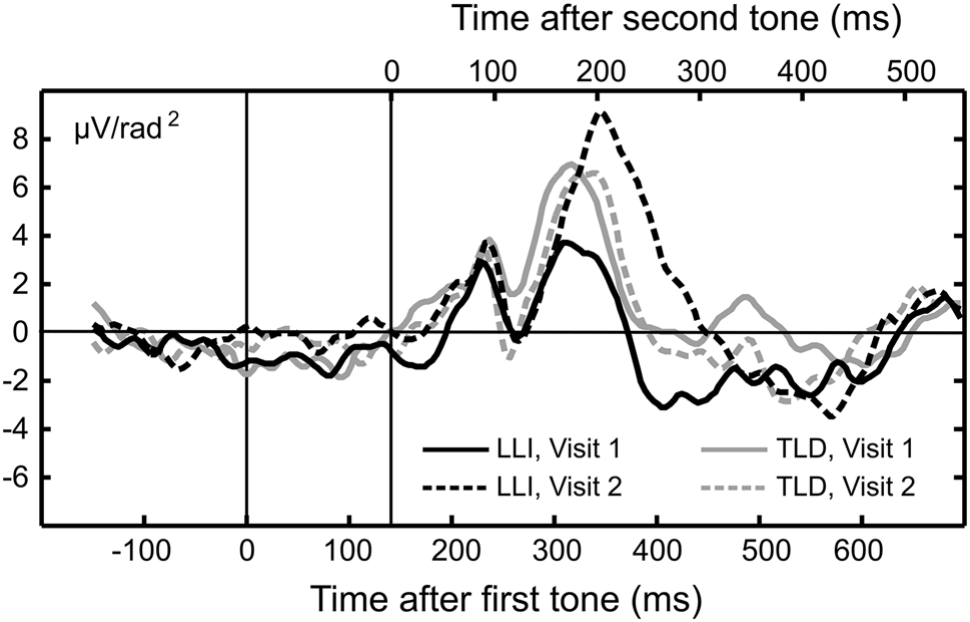
Grand mean CSD difference waveforms [∆ (deviant − standard)] over a representative group of fronto-central sensors, including Fcz and their posterior neighbors 5 and 55, at Visit 1 (*solid lines*) and Visit 2 (*dashed lines*) for the two groups in the study, 12 children with TLD (*gray lines*) and 21 children with LLI (*black lines*). The bottom abscissa indicates the time scale with respect to the first tone in a doublet, the top abscissa the time scale with respect to the second tone. Children with LLI showed a delayed difference wave in the downward slope of the P2 post-intervention, with latencies at Visit 2 and Visit 1 amounting to 284 and 224 ms, respectively, following onset of the second tone in a pair. No systematic latency change evinced in the TLD group

Figure 7 illustrates the CSD difference waveforms averaged across a representative group of fronto-central sensors, including electrode site Fcz and their nearest posterior neighbors 55 and 5. Jackknife *t* tests conducted for the latency of peaks (defined as the time point when the voltage reached 50 % of peak amplitude) in the difference waveforms [∆ (deviant − standard)] showed that the difference wave in the P2 range (peaking around 200 ms after the second tone) was significantly delayed only in the LLI group, when comparing Visit 1 and Visit 2. As evident in Fig. 2, this effect was reflective of the downward slope of the P2 being significantly extended in time, but only for the deviant stimulus, at Visit 2, and in the LLI group: The 50 % amplitude point of the downward slope of the resulting difference wave in the P2 range was 284 ms at Visit 2 and 224 ms at Visit 1, with latencies relative to the onset of the second stimulus of a tone pair, *t*(20) = 4.67, *p* < 0.001. No systematic latency change was observed for any of the other components, or in the TLD group.

## Discussion

The present research set out to explore the electrophysiological correlates of processing rapid tone sequences (pairs) in children with LLI and controls with TLD, in the context of a training intervention administered in the LLI group. Comparing the ERP waveforms for standard and deviant tone pairs across two visits in each of the two groups, we found that both LLI and TLD individuals displayed pronounced sensitivity to deviant sound information early in auditory cortical processing, as measured relative to the second of two tones in a pair: Early ERP components of P1 and N1 reliably discriminated between standard and deviant tone doublets, across visits and groups. Effects of repeated measurement were seen for the subsequent MMN deflection (160–220 ms): It displayed a fronto-lateral CSD topography as observed in previous research (Martin et al. 2003), and did not differ between TLD and LLI children, but showed amplitude increase (greater deviant-standard difference) from Visit 1 to Visit 2, across groups. By contrast, the later P2 component reflective of widespread electrocortical communication showed changes in standard-deviant discrimination across visits, specifically in the LLI group: The late portion of the P2 (264–280 ms) was selectively enhanced to the deviant stimulus in children with LLI after training. This amplitude difference was accompanied by a distinct latency shift of the deviant-standard difference waveform, in LLI children at Visit 2. Thus, the present findings may be taken to indicate a change in neurocognitive processing of deviant sounds selectively in the LLI group. Given its group and time selectivity, this change may be associated with the intervention in which remediation induces heightened sensitivity to differences between standard and deviant tone pairs, paralleling the behavioral benefits on language tasks that were also observed in this study. Since the present design is not a randomized clinical trial, there are many additional alternative causal mechanisms that may lead to interaction effects observed here. Specifically, because no intervention was given to the TLD group, the question may arise if the selective electrophysiological changes in the LLI group reflect (1) an a priori group difference in reactivity, or (2) mean differences on CELF, such as that the LLI children had more potential for gain in language functioning, with TLD children at ceiling. Although the first concern cannot be ruled out given the weaknesses of the present experimental design, a priori (i.e., at the first visit) differences between groups were specific to the standard tone pair, but cross-visit effects in LLI children were specific to the deviant stimulus. Similarly, the study by Heim et al. (2013) did not show evidence of systematic a priori reactivity in cortical responsiveness when considering early oscillatory activity. Regarding the second concern, examining the distribution of CELF values in the LLI and TLD groups showed no evidence of the TLD children being at ceiling, and no difference between the groups regarding the overall variability. Amplitude and latency measures for the late aspect of the P2 component converged in the present study, showing pronounced standard-deviant differences at Visit 2, for the LLI group only. Various authors have discussed heightened latency and longer duration of late positive components as potential indices of effortful processing, the duration of cognitive processing, the allocation of attentional resources, and changes in cognitive strategies used to approach a given task (McCarthy and Donchin 1981; for reviews see Crowley and Colrain 2004; Polich 2007; Steinmann et al. 2011). Although an attribution of the P2 effects seen in the present report to one specific cognitive process is not possible, this body of literature suggests that LLI children responded differently to deviant tone doublets after training, and they did so at a late temporal stage.

The fact that the later, but not early ERP components were impacted may point to a temporal locus of training-related effects in downstream processing, persisting beyond early sensory processes. These findings complement results on sensory oscillatory activity in the same sample of children, reported in a previous paper (Heim et al. 2013). In that study, pronounced group differences in early evoked gamma oscillations were observed specifically in response to the second tone of a pair. Early oscillations were affected by the training such that following completion of the protocol, group differences in evoked gamma amplitude were diminished. These focal, early effects do not map linearly onto the current results, in which groups diverged in terms of later electrocortical activity after the LLI children underwent remediation. One obvious interpretation is that the late increase in P2 amplitude reflects recruitment of processes not used or not available prior to intervention, aiding in parsing the sequences and/or detecting patterns of auditory change. This interpretation is in line with mechanisms proposed as mediators of beneficial effects of LLI intervention: The FFW program applied here was designed to develop children’s foundational cognitive skills, essential for fostering elementary school language and reading ability (http://www.scilearn.com/). As documented on the manufacturer’s website, the basic FFW protocol, when implemented in scholastic environments, may lead to improved annual student assessment scores in areas beyond English language arts, including mathematics and reasoning.

As mentioned in the Introduction, meta-analytic work on FFW (Strong et al. 2011) has not supported its effectiveness for treating language problems. For instance, general language gains after FFW have been shown to not exceed the efficacy of one-on-one speech therapy or academic enrichment provided for a comparable amount of time (Gillam et al. 2008). The current study explores changes in large-scale electrocortical activity that accompany FFW training, used here for its potential to induce neural changes in the course of intervention: There is evidence that LLI children (and to a lesser extent typically developing controls) benefited from this training with respect to ERP activity underlying selective auditory attention in a story-listening context (Stevens et al. 2008). In dyslexia, Temple et al. (2003) reported increased activity in the anterior cingulum cortex and hippocampal region during pseudoword decoding upon completion of FFW, leading them to speculate on training-related alterations in attentional and memory mechanisms.

To the extent that changes in attention focus and/or attention control may be promoted by the FFW intervention, it is notable that the auditory P2 in response to the second tone of a pair showed pronounced effects in the current study. Given its latency between 264 and 280 ms, this component is considered outside the window of initial sensory analysis, and has often been reported in the context of research on auditory selective attention and auditory working memory. P2 amplitude enhancement as found here has been traditionally seen when attending to auditory targets (e.g., Picton and Hillyard 1974), or when auditory stimuli match the target item in a working memory task (e.g., Alain et al. 2009). Thus, the present pattern of results would be compatible with a change in cognitive control strategies towards rapid auditory stimulus sequences, specifically in LLI children after training. A related and unexpected finding points in the same direction: Pronounced latency differences were observed in the P2 range, with greater amplitude in LLI children following intervention linked to delayed latency of the subsequent negative-going deflection in the difference waveform. It is appealing to consider these two variations (P2 amplitude increase and subsequent latency delay) as amalgamated facets of altered neurocognitive processing, both reflective of changes in strategic—and potentially effortful—control, after training, in children with LLI.

We observed an interaction effect of Visit by Stimulus in a time window associated with the MMN response (e.g., Näätänen et al. 2007), after the obligatory P1 and N1 (Ruhnau et al. 2011), at latencies of 160–220 ms. This response showed a polarity reversal of the CSD maps bilaterally, over lateral fronto-temporal electrodes, with positivity at frontal and negativity at temporal sites. Although this CSD topography deviates from many reports on voltage difference topographies, previous work using CSD maps has observed similar patterns of lateral (temporal) polarity reversal (Martin et al. 2003)—not inconsistent with the present CSD map. Future research may assess the robustness of this source density configuration. Compatible with an interpretation of this effect as the MMN, it consisted of a negative difference waveform, more negative after deviant versus standard tone doublets. This difference waveform showed heightened negativity at Visit 2 compared to Visit 1. In the present work, the amplitude of the MMN-like deflection was not modulated by LLI status, which may be seen as at odds with studies employing rapidly presented sounds (cf., Bishop 2007). The fact, however, that we used tone pairs instead of single stimulus trains of deviants and standards may assist in explaining this notable absence of interaction effects with participant group. For instance, superposition of the individual ERP responses to both stimuli of a pair may affect the typical MMN response and topography, potentially changing its sensitivity to a subset of neural generators contributing to the MMN scalp potential. Moreover, the response to the first of two tones (which is always the same), may be unaffected by group or specific treatment (Heim et al. 2013) and thus diminish the proportion of the signal that is being modulated by inter-individual differences or training.

There were very few ERP differences between groups during the first assessment visit, which merits discussion in this context as well. First, a host of studies have shown group differences between LLI and TLD children on a variety of ERP indices (e.g., Bishop et al. 2007, 2012; Näätänen et al. 2014). Second, the previous study from our laboratory also observed pronounced differences in sensory evoked gamma power at the baseline measurement (Visit 1). As discussed above, however, the present ERP waveforms to a large extent reflect overlapping processes in response to both stimuli of the doublet, including the first stimulus, for which we did not find any group differences in the Heim et al. (2013) study. In addition, the strength of the present research design arises from repeated measurement times, i.e., by including pre-post changes in the statistical model, but the sample size may be too limited to detect single-session group differences that may be subtle and highly variable in nature. In line with this notion and as mentioned in the Introduction, previous reports of electrophysiological differences in smaller samples of LLI and TLD children tend to show variability across laboratories, which may be due to differences in experimental designs, but also in the variability of sample selection, often complicated by the existence of comorbidity of LLI with other developmental disorders, as well as behavioral and emotional disturbances (Heim and Benasich 2006; Tallal and Heim 2015). The present study included children with a relatively wide range of language problems, with a few LLI individuals performing at the low average spectrum in some CELF subtests. Although such mild impairment renders a young student to struggle with academic tasks, future work may want to replicate this finding with a sample of children, selected to be more severely affected across multiple areas of language function.

This leads one to consider an important difference between the ERP technique and measures of oscillatory activity: The strength of the ERP method is in the high-fidelity representation of neurocognitive processes time-locked to the onset of the tone pairs. This strength was leveraged here by evaluating effects of the experimental design for a sequence of deflections, representing a cascade of temporally unfolding neural events. Tone pairs were used because rapid sequence processing has been discussed as a key aspect of auditory language processing (e.g., Choudhury and Benasich 2011; Hari and Renvall 2001; Tallal and Gaab 2006). One problem of ERPs in response to rapid stimulus pairs lies in the potential for superposition of deflections elicited by the members of the doublet. At a temporal distance of 70 ms, it is for instance conceivable that the P2 response to the first stimulus partly overlaps with the P1/N1 of the second stimulus, introducing distortions of the known auditory ERP morphology that may make interpretation difficult. Here we addressed this problem by examining two types of tone pairs and by considering both the difference as well as non-difference waveforms. This approach helped us to identify temporal regions in which electrophysiological differences between groups and/or visits emerged.

As an important methodological step, we used the CSD transformation of the scalp voltage data for all analyses. This reference-free representation assisted in reducing blurring of the voltage map due to volume conduction and as a consequence facilitated our efforts in assessing the cortical physiology in terms of latency and amplitude across two groups of children and two measurement times. While outside the scope of the present paper, CSD scalp topographies showed considerable inter-individual consistency, and a quantitative comparison between CSD and voltage maps in terms of reliability in pediatric samples may be an interesting goal for future research.

In summary, the current study demonstrates that neurocognitive processes beyond initial sensory analysis were substantially altered in children with LLI when comparing measurements taken before and after a training intervention was given. These effects had widespread topographical distribution and were defined by an increased response to tone pair stimuli deviating from a standard pattern. Thus, we conclude that processing of tone sequences is altered after training, potentially as a consequence of LLI children adopting compensatory cognitive strategies such as selective attention, or working memory.
